# Development and characterization of potential larvicidal nanoemulsions against *Aedes aegypti*

**DOI:** 10.3762/bjnano.15.10

**Published:** 2024-01-18

**Authors:** Jonatas L Duarte, Leonardo Delello Di Filippo, Anna Eliza Maciel de Faria Mota Oliveira, Rafael Miguel Sábio, Gabriel Davi Marena, Tais Maria Bauab, Cristiane Duque, Vincent Corbel, Marlus Chorilli

**Affiliations:** 1 Department of Drugs and Medicines, School of Pharmaceutical Sciences, São Paulo State University (UNESP), Araraquara, São Paulo, Brazil; 2 Departamento de Ciências Biológicas e da Saúde, Universidade Federal do Amapá, Macapá, AP, Brazil; 3 Department of Biological Sciences, São Paulo State University (UNESP), School of Pharmaceutical Sciences, Campus Araraquara, São Paulo, Brazil; 4 Department of Preventive and Restorative Dentistry, Araçatuba Dental School - São Paulo State University (UNESP), Araçatuba, SP, Brazil; 5 Institut de Recherche pour le Développement (IRD), MIVEGEC, Univ. Montpellier, CNRS, IRD, 911 Av Agropolis, 34 394 Montpellier, France; 6 Fundação Oswaldo Cruz (FIOCRUZ), Instituto Oswaldo Cruz (IOC), Laboratório de Fisiologia e Controle de Artrópodes Vetores (Laficave). Avenida Brasil, 4365 Manguinhos, Rio de Janeiro – RJ, CEP: 21040-360, Brazil

**Keywords:** colloidal stability, drug delivery system, hydrophile–lipophile balance, monoterpenes

## Abstract

Plant-based insecticides offer advantages such as negligible residual effects, reduced risks to both humans and the environment, and immunity to resistance issues that plague conventional chemicals. However, the practical use of monoterpenes in insect control has been hampered by challenges including their poor solubility and stability in aqueous environments. In recent years, the application of nanotechnology-based formulations, specifically nanoemulsions, has emerged as a prospective strategy to surmount these obstacles. In this study, we developed and characterized nanoemulsions based on cymene and myrcene and assessed their toxicity both in vitro using human keratinocytes (HaCAT) cells and in an in vivo model involving *Galleria mellonella* larvae. Additionally, we investigated the insecticidal efficacy of monoterpenes against the mosquito *Aedes aegypti*, the primary dengue vector, via larval bioassay. Employing a low-energy approach, we successfully generated nanoemulsions. The cymene-based nanoemulsion exhibited a hydrodynamic diameter of approximately 98 nm and a zeta potential of −25 mV. The myrcene-based nanoemulsion displayed a hydrodynamic diameter of 118 nm and a zeta potential of −20 mV. Notably, both nanoemulsions demonstrated stability over 60 days, accompanied by controlled release properties and low toxicity towards HaCAT cells and *Galleria mellonella* larvae. Moreover, the nanoemulsions exhibited significant lethality against third-instar *Aedes aegypti* larvae at a concentration of 50 mg/L. In conclusion, the utilization of nanoemulsions encapsulating cymene and myrcene presents a promising avenue for overcoming the limitations associated with poor solubility and stability of monoterpenes. This study sheds light on the potential of the nanoemulsions as effective and environmentally friendly insecticides in the ongoing battle against mosquito-borne diseases.

## Introduction

*Aedes aegypti* (Linnaeus, 1762) is a mosquito species that is cosmopolitan and well adapted to anthropized and peridomestic environments. It is an important vector of arboviruses, including dengue, chikungunya fever, zika, and urban yellow fever and can cause alarming socio-economic impacts in the affected regions [1]. The World Health Organization (WHO) considers dengue, zika, and chikungunya as neglected and emerging tropical diseases transmitted by mosquitoes and as one of the main concerns in developing countries, which may become a major public health problem worldwide. This problem is evidenced by recent cases of Zika virus infection in Brazil and their relationship with microcephaly in newborns [2]. In the case of dengue, the most prevalent viral infection transmitted by *Aedes* mosquitoes with clinical forms ranging from asymptomatic to fatal cases, around 3.9 billion people in more than 129 countries are at risk [3]. The continuous and indiscriminate use of synthetic insecticides for the control of the *Aedes aegypti* mosquito (Linnaeus) has been responsible for the emergence of insecticide-resistant mosquitoes [4–5]. Therefore, it becomes urgent to search for safer and more effective vector control agents to prevent vector-borne diseases [6].

Bioinsecticides from plant derivatives, which degrade rapidly in the environment and have less toxicity in non-target organisms, are a promising option for vector control [7]. Terpenes are the largest group of secondary plant metabolites and have shown promising health benefits as antioxidant and anti-inflammatory agents in many animal studies [8–9]. The compound *p*-cymene, also known as *p*-cymol or *p*-isopropyltoluene, is a monocyclic hydrocarbonated monoterpene naturally occurring in essential oils (EOs) of various aromatic plants, including the genera *Artemisia*, *Protium*, *Origanum*, and *Thymus*. Myrcene is an acyclic monoterpene found in hops, lemongrass, basil, and mangos [10].

Some intrinsic characteristics of monoterpenes, mainly poor water solubility and high volatility, make their formulation a true challenge. In this regard, nanoemulsions (NEs), which are dispersions of two immiscible liquids with one of them dispersed as small droplets [11–12], stand out as new delivery vehicles for these bioactive compounds. They are especially important to enhance the water availability of poorly water-soluble compounds, which is achieved when the oil constitutes the internal phase. In this case, oil-in-water nanoemulsions or aqueous nanoemulsions are obtained. The main advantage of NEs is their better kinetic stability compared to macroemulsions. Also, the NEs protect the EO constituents from oxidation, in addition to promoting better sensorial properties [13]. Moreover, the development of aqueous nanoemulsions would enable a better dispersion of vector control agents, inducing a controlled release and a possibly higher effectiveness in eliminating immature stages of mosquitoes [14].

NEs can be obtained through two general approaches, that is, high-energy methods and low-energy methods. The high-energy methods are characterized by using equipment such as sonicators, high-speed homogenizers, and high-pressure homogenizers, which provide high energy input during processing, leading to the generation of dispersed material on a nanoscale [15]. The low-energy methods are characterized by the use and control of the chemical energy of the system in the formation of droplets on the nanoscale. A crucial point is that these systems can be obtained at low cost and with eco-friendly techniques [16–17].

Griffin established the hydrophile–lipophile balance (HLB) as a tool for classifying and selecting non-ionic emulsifiers [18]. The determination of the required HLB (rHLB) of essential oils appears as a critical step for the development of stable emulsions [19]. Determining the required HLB, one can obtain the nanoemulsion with the smallest droplet size, leading to more stable formulations [20]. The rHLB is usually determined by preparing NEs with different ratios of surfactant blends and choosing the most stable formulation to determine the rHLB of the oil phase [21].

Biocompatibility assessment is an essential aspect of the development of NEs, particularly for biomedical and cosmetic applications, as it determines the safety and efficacy of the formulations. The assessment involves evaluating the potential cytotoxicity and genotoxicity of the NEs on different cell types and determining the effect on the immune response in vivo. In vitro cytotoxicity assays are an important tool for evaluating the safety of NEs. HaCaT cells are a widely used human keratinocyte cell line that exhibits several characteristics of normal human epidermal keratinocytes, making them an excellent model for evaluating cytotoxicity [22].

In vivo toxicity studies are also crucial for evaluating the safety of NEs. *Galleria mellonella* larvae have emerged as an alternative to mammalian models for in vivo studies of acute toxicity because of their low maintenance cost, easy handling, and high similarity in immune response with mammals. Furthermore, *G. mellonella* larvae have been successfully used to evaluate the acute toxicity of various nanoparticles and drugs [23]. The immune response of *G. mellonella* larvae can be evaluated by monitoring their survival rate and melanization response [24].

The aim of the present work was (i) to develop stable oil-in-water nanoemulsions containing myrcene or cymene as the dispersed phase, (ii) to determine the required rHLB values for emulsion stability, (iii) to assess the biocompatibility via in vitro and in vivo assays, and (iv) to evaluate the bioefficacy of the NE against *Aedes aegypti* mosquito larvae.

## Results and Discussion

### Preparation and characterization of the nanoemulsions

The determination of the required HLB (rHLB) is an important step in the development of NEs containing volatile oils [19,25]. From the determination of the rHLB, it is possible to determine the best ratio between two surfactants, one more lipophilic and one more hydrophilic, which will be necessary to obtain a stable NE [18]. The rHLB of myrcene and cymene was determined using a mixture of Span 80 (lipophilic) and Tween 20 (surfactant). At a time of 24 h after preparation, formulations containing cymene with HLB values of 10–13 showed a yellowish layer on the surface, which may be an indication of phase separation (Figure S1, Supporting Information File 1). Formulations with HLB values of 16 and 16.7 showed a milky appearance and slight creaming, which may be indicative of Ostwald maturation, a very important mechanism when it comes to the instability of NEs. It is related to the difference between the droplet sizes in the formulation, with the smaller droplets having greater chemical potential and, thus, diffusing to the larger ones [26]. Formulations with HLB values of 14 and 15 were the ones that presented the best visual characteristics, in addition to a bluish appearance, a characteristic of NEs [27–28]. Thus, the formulations with HLB values of 14 and 15 were selected for analysis by DLS.

After 24 h (D1), the formulation with HLB 14 had a droplet size of 116 ± 0.40 nm, and after 21 days there was no significant change in particle size, nor in polydispersity index (PdI) and zeta potential. The formulation with HLB 15 exhibited smaller particle size and lower PdI and zeta potential than the HLB 14 formulation. Also, there was no significant variation in these parameters throughout the analyzed period (60 days) (Table 1). For this reason, the formulation with HLB 15 was the formulation chosen as the rHLB of cymene.

**Table 1:** Hydrodynamic diameter, PdI, and zeta potential of Cym-NEs.^a^

	HLB 14			HLB 15		
	
Time	Size (nm)	PdI	Zeta potential (mV)	Size (nm)	PdI	Zeta potential (mV)

D1	116.0 ± 0.40	0.322 ± 0.024	−34.7 ± 1.1	98.46 ± 0.83	0.209 ± 0.002	−25.9 ± 0.43
D7	111.2 ± 1.58	0.285 ± 0.007	−36.1 ± 0.7	96.74 ± 1.00	0.226 ± 0.006	−24.3 ± 0.80
D14	107.6 ± 1.59	0.331 ± 0.023	−26.8 ± 0.4	95.43 ± 1.20	0.204 ± 0.006	−25.4 ± 1.45
D21	106.5 ± 0.73	0.350 ± 0.003	−34.5 ± 0.8	98.7 ± 1.508	0.216 ± 0.004	−25.5 ± 0.68
D30	—	—	—	96.09 ± 0.61	0.218 ± 0.009	−23.3 ± 0.45
D45	—	—	—	97.69 ± 0.20	0.205 ± 0.013	−25.5 ± 1.14
D60	—	—	—	89.70 ± 0.17	0.240 ± 0.004	−25.9 ± 0.35

^a^The data are expressed as mean ± standard deviation, *n* = 3.

After 24 h, the myrcene formulations with lower HLB values (10–11), that is, a greater amount of the surfactant (Tween 20) plus lipophilic (Span 80), showed classic signs of instability (i.e., creaming) (Figure S2, Supporting Information File 1). Formulations with HLB values of 12–14 and 16.7 showed a milky appearance and a more viscous appearance, characteristic of emulsions with droplets on the micrometric scale. It is important to mention that these formulations also showed signs of instability after 21 days. The formulations with HLB 15 and 16 were the ones that presented the best visual appearance, such as a bluish appearance characteristic of nanoemulsions, and maintained these characteristics over time. Thus, these formulations were selected for DLS.

The droplet size and PdI of the formulation with HLB 16 were slightly smaller than those of the formulation with HLB 15 (Table 2). Over time, there was no significant variation in the size for both formulations. Unlike the formulation containing cymene, the best formulation with myrcene was the one with HLB 16, which has in its composition a greater amount of Tween 20, the more hydrophilic surfactant.

**Table 2:** Hydrodynamic diameter, PdI and zeta potential of Myr-NEs.^a^

	HLB 15				HLB 16	

Time	Size (nm)	PdI	Zeta potential (mV)	Size (nm)	PdI	Zeta potential (mV)

1D	123.9 ± 1.15	0.369 ± 0.02	−17.4 ± 0.0	118.8 ±1.2	0.241 ± 0.01	−21.1 ± 0.3
7 D	113.5 ± 1.45	0.352 ± 0.05	−20.7 ± 0.3	118.0 ± 3.7	0.227 ± 0.006	−21.5 ± 0.5
14 D	112.0 ± 0.51	0.364 ± 0.01	−26.8 ± 0.4	110.8 ± 3.4	0.235 ± 0.007	−22.6 ± 2.3
21 D	115.4 ± 0.45	0.240 ± 0.02	−24.3 ± 0.5	104.5 ± 0.4	0.255 ± 0.005	−25.5 ± 0.6
D30	—	—	—	105.5 ± 0.7	0.227 ± 0.009	−25.3 ± 1.63
D45	—	—	—	99.93 ± 1.45	0.246 ± 0.012	−21.0 ± 2.47
D60	—	—	—	84.50 ± 0.82	0.217 ± 0.008	−20.7 ± 0.95

^a^The data are expressed as mean ± standard deviation, *n* = 3.

It has been shown that a significant difference of the headgroup size of the surfactants has a synergistic effect on emulsion stabilization. Furthermore, the use of mixed surfactants enhances the properties of the interfacial film, leading to improved adsorption between the oil and water phases and enhancing the stability of nanoemulsions [29].

Previous studies have reported similar findings regarding NEs containing terpenes. Polydispersity index values comparable to those observed in our study were obtained, indicating the formation of stable and suitable NEs for larvicidal applications [30]. It is noteworthy that several studies have utilized essential oils containing chemical components structurally analogous to terpenes, thus, achieving table formulations using the same surfactants and active ingredient concentration (5%) [26–28]. However, there is only a limited number of studies that focus specifically on the production and characterization of nanoemulsions incorporating cymene or myrcene. Nevertheless, it has been demonstrated that a high-energy method can yield a nanoemulsion comprising 5% *p*-cymene stabilized with 1% Tween 80, with droplet sizes measuring approximately 150 nm, which maintained its stability for 60 days [31].

The zeta potential is used to predict the stability of dispersions, and its value depends on the physicochemical properties of active ingredients, polymers, vehicles, and the presence of electrolytes and their adsorption [32]. The zeta potential values found for the NEs obtained remained stable in the analyzed period, which indicates the stability of the formulation to avoid Ostwald maturation and coalescence of the droplets. Similar zeta potential characteristics, between 20 and 30 mV, have been described in other studies about nanoemulsions containing terpenes suitable for larvicidal applications [30,33–34].

Regarding the physical characterization, the bluish reflex is characteristic of this type of colloidal system, and it is attributed to the Tyndall effect, making it a valuable macroscopic indicator of nanodroplet generation [16]. In addition, Forgiarini et al. indicated that a suitable nanoemulsion should have small drops of the dispersed phase (average below 300 nm) [35]. Izquierdo et al. stated that polydispersion index values close to 0.2 are an indication of kinetic stability with an almost monomodal distribution [36]. Thus, considering that in this study the stable formulations had similar size distribution profiles and low polydispersity index, the present study on cymene and myrcene nanoemulsions may be considered promising.

### Nanoparticle tracking analysis

From the two results obtained above, the HLB 15 formulation containing cymene and the HLB 16 formulation containing myrcene underwent nanoparticle tracking analysis (NTA). NTA is a technique for direct and real-time visualization, sizing, and counting of nanometric materials suspended in aqueous media [37]. According to NTA measurements, the Cym-NE particle size was 145.7 ± 7.7 nm, while the Myr-NE particle size was 126.4 ± 5.6 nm, confirming the nanometric droplet size.

#### Cryogenic transmission electron microscopy

**Figure 1:**
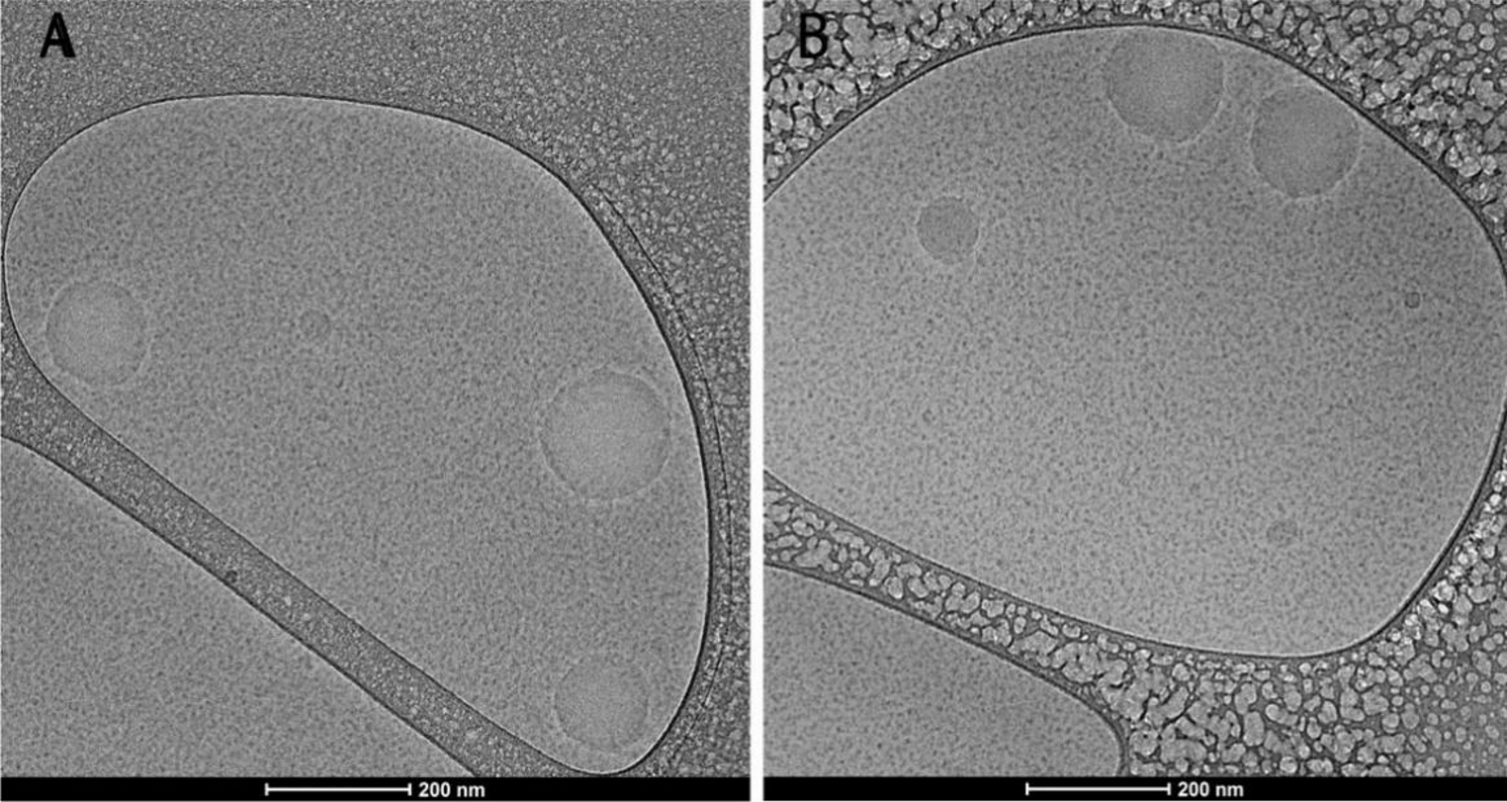
Cryogenic transmission electron microscopy of (A) Cym-NE and (B) Myr-NE.

Cryogenic transmission electron microscopy (cryo-TEM) is one of the most useful techniques for the investigation of NEs, since it provides detailed information about the internal structure of colloidal systems observed in their native state [38]. In cryo-TEM, it was possible to observe spherical droplets (Figure 1). Similar results of spherical droplets smaller than 180 nm were observed with cryo-TEM [39]. This technique is widely used to characterize the morphology of nanoemulsions and faithfully confirms the results obtained with other techniques [40].

#### In vitro drug release

**Figure 2:**
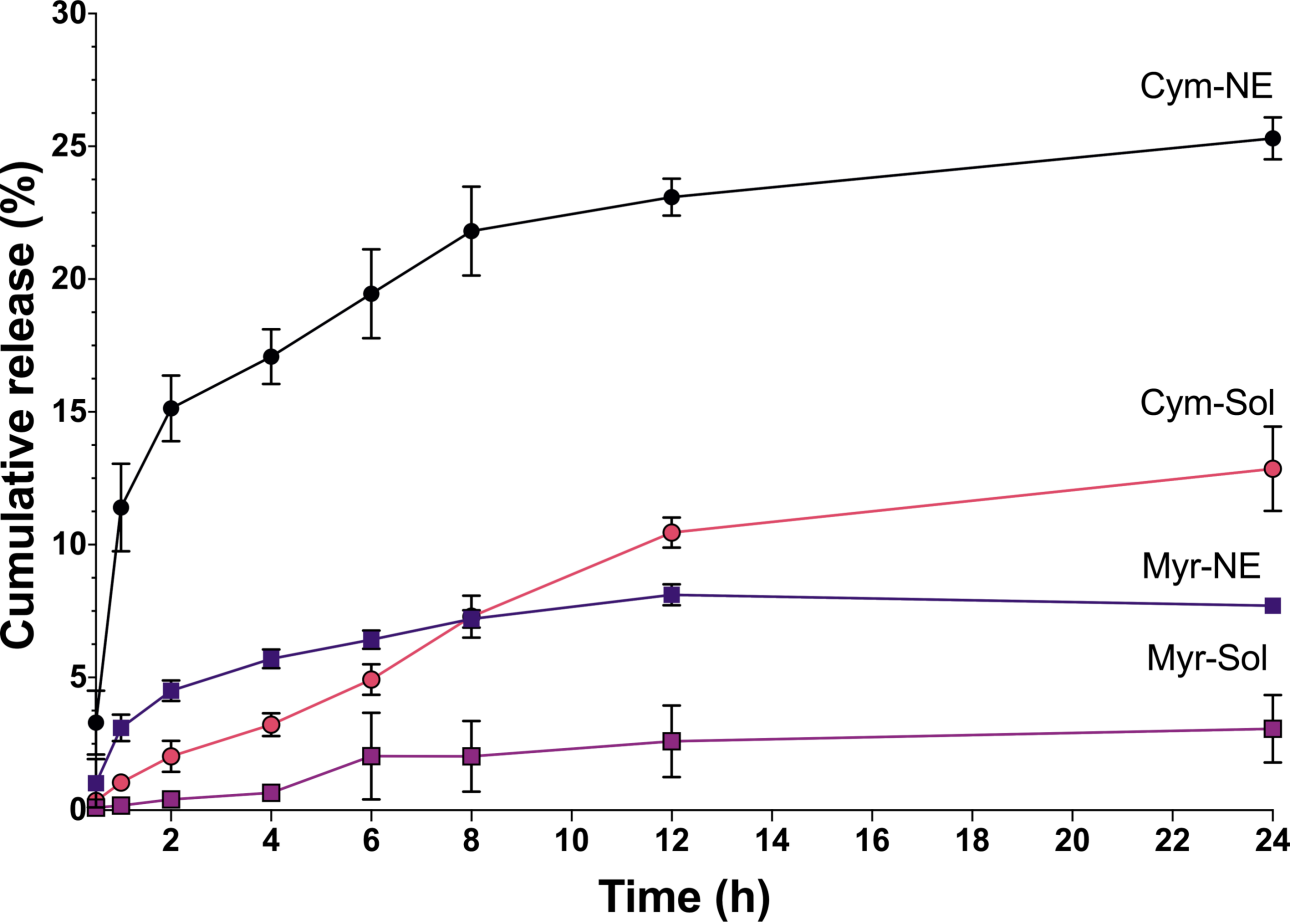
The in vitro drug release of nanoemulsions (Cym-NE, Myr-NE) and free terpenes (Cym-Sol and Myr-Sol).

One potential advantage of using NEs is their ability to enhance drug solubility and bioavailability. NEs have been shown to increase the solubility of poorly soluble drugs, such as monoterpenes, which can improve drug delivery and efficacy. The cumulative release of both free terpenes was lower than the cumulative release of nanoemulsions (Figure 2).

The observed differences in the release of terpenes can be attributed to their specific chemical characteristics. Cymene has a lower log *P* value than myrcene, indicating higher water solubility. This greater solubility in water may contribute to its higher release rate when compared to myrcene. Additionally, cymene has a lower molar volume than myrcene, which could also enhance its release rate (Table 3).

**Table 3:** In silico molecular/physicochemical properties of cymene and myrcene.

Compound	log *P*	Molar volume (cm^3^)	Water solubility (mg/L)

cymene	4.02	155.8 ± 3.0	27.88
myrcene	4.58	177.0 ± 3.0	6.923

Previous research supports the fact that monoterpenes exhibit slower release than other drugs from delivery nanosystems [41]. This phenomenon can be explained by the higher surfactant/oil solubility, which leads to a stronger affinity to the oil–surfactant core–shell structure within the micelles. Consequently, a lower amount of monoterpenes is released into the surrounding medium [42].

Among the mathematical models used to study drug kinetics, the Korsmeyer–Peppas release model proved to be the most suitable for our formulations (Table 4). Our results show that Cym-NE has a *k* value of 10.4, while Myr-NE has a *k* value of 3.3. A higher *k* value indicates faster drug release, while a lower *k* value indicates slower transport kinetics and, consequently, poor drug release from nanocarriers [43].

**Table 4:** Mathematical release kinetics models for nanoemulsions (Cym-NE, Myr-NE) and free terpenes (Cym-Sol and Myr-Sol).

	Model	Zeroth order	First order	Higuchi	Hixson–Crowel	Korsmeyer–Peppas

Cym-Sol	*k*	0.0001	0.0069	24.783	0.0023	1.5587
	*R* ^2^	0.8946	0.881	0.8993	0.8729	0.9534
	*n*	—	—	—	—	0.6865
Cym-NE	*k*	0.0008	0.0191	6.5154	0.0059	10.465
	*R* ^2^	0.6729	0	0.6161	0	0.8776
	*n*	—	—	—	—	0.3005
Myr-Sol	*k*	0	0.0017	0.6424	0.0006	0.4836
	*R* ^2^	0.857	0.7223	0.8576	0.7195	0.8813
	*n*	—	—	—	—	0.6152
Myr-NE	*k*	0	0.0053	2.1477	0.0017	3.3949
	*R* ^2^	0.6317	0	0.6145	0	0.8419
	*n*	—	—	—	—	0.3079

Furthermore, both Cym-NE and Myr-NE demonstrated a transport exponent value (*n*) of 0.3, indicating a release mechanism primarily driven by Fickian diffusion [44]. The free terpenes exhibited a value of 0.6, suggesting an anomalous transport mechanism for drug release. This mechanism involves a combination of diffusion and dissolution processes for drug release [45].

These results indicate that the use of NEs may be an effective strategy to improve the control of the release rate of terpenes for more durable and effective control of immature stages of pest vectors.

#### Larvicidal properties of NEs against *Aedes aegypti*

The potential larvicidal activity of free monoterpenes and nanoemulsions was assessed using third-instar *Aedes aegypti* larvae. The negative control group was treated with surfactant solutions (Span 80 and Tween 20) at the same concentrations as in the nanoemulsions. Mortality of mosquito larvae was recorded after 24 h of exposition according to the WHO protocol [55].

Free cymene exhibited a concentration-dependent larvicidal activity. At 5 mg/L, mortality was 20% ± 4%, rising to 83% ± 2.3% at 25 mg/L and peaking at 98.6% ± 2.3% at 50 mg/L. Surprisingly, the cymene NE displayed a slightly reduced efficacy at lower concentrations (5 mg/L and 25 mg/L) compared to free cymene. This suggests that the encapsulation influences the bioactivity, potentially because of improved dispersion and controlled release of cymene.

Similarly, free myrcene exhibited a concentration-dependent efficacy. Myrcene NEs consistently outperformed free myrcene at all concentrations, indicating a better dispersion of the nanoemulsions in aqueous media. This was most prominent at lower concentrations, resulting in mortality rates of 10.6% ± 2.3% at 5 mg/L and up to 100% at 50 mg/L (Table 5).

**Table 5:** Average mortality of *Aedes aegypti* larvae after 24 h of exposure to the free monoterpenes and their nanoemulsion.

	Average mortality (%) after 24 h		

	5 mg/L	25 mg/L	50 mg/L
	
Cym-free	20 ± 4	83 ± 2.3	98.6 ± 2.3
Cym-NE	14.6 ± 2.3	78.6 ± 4.6	100 ± 0
Myr-free	13.3 ± 2.3	81.3 ± 4.6	98.6 ± 2.3
Myr-NE	10.6 ± 2.3	94.6 ± 2.3	100 ± 0

#### Cytotoxicity of NEs in human keratinocytes

The evaluation of the biocompatibility in human cells is an important step in the development and commercialization of any drug [46]. Here, the toxicity of the terpene-based formulations was evaluated in the HaCAT cell line (Table 6). The results show that the IC_50_ values of the free terpenes were lower those of the nanoemulsions, suggesting that the nanoemulsification reduces the cytotoxicity of terpenes. It is important to note that the surfactant solutions presented the highest IC_50_ values, which indicates that the composition of the nanoemulsion may influence its ability to decrease terpene toxicity. These results are in line with the existing literature, which indicates that monoterpenes exert low cytotoxicity on keratinocyte cells, either free [47–49] or in nanoemulsions [50–51]. It is important to highlight that the excipients used in the formulation are within the maximum concentration recommended by the FDA (7% for Span 80 and 5% for Tween 20) [52].

**Table 6:** Inhibitory concentrations 50 (IC_50_) of the NEs (Cym-NE and Myr-NE), free terpenes (Cym-free and Myr-free), and surfactants solution (B-Cym and B-Myr) in human keratinocytes.

	IC_50_ (mg/mL)

Cym-free	6.43 ± 0.56
Myr-free	1.86 ± 0.15
B-Cym	16.98 ± 0.90
B-Myr	22.37 ± 0.32
Cym-NE	14.95 ± 0.64
Myr-NE	2.37 ± 0.33

#### Acute toxicity of LNCs in alternative in vivo model using *Galleria mellonella*

**Figure 3:**
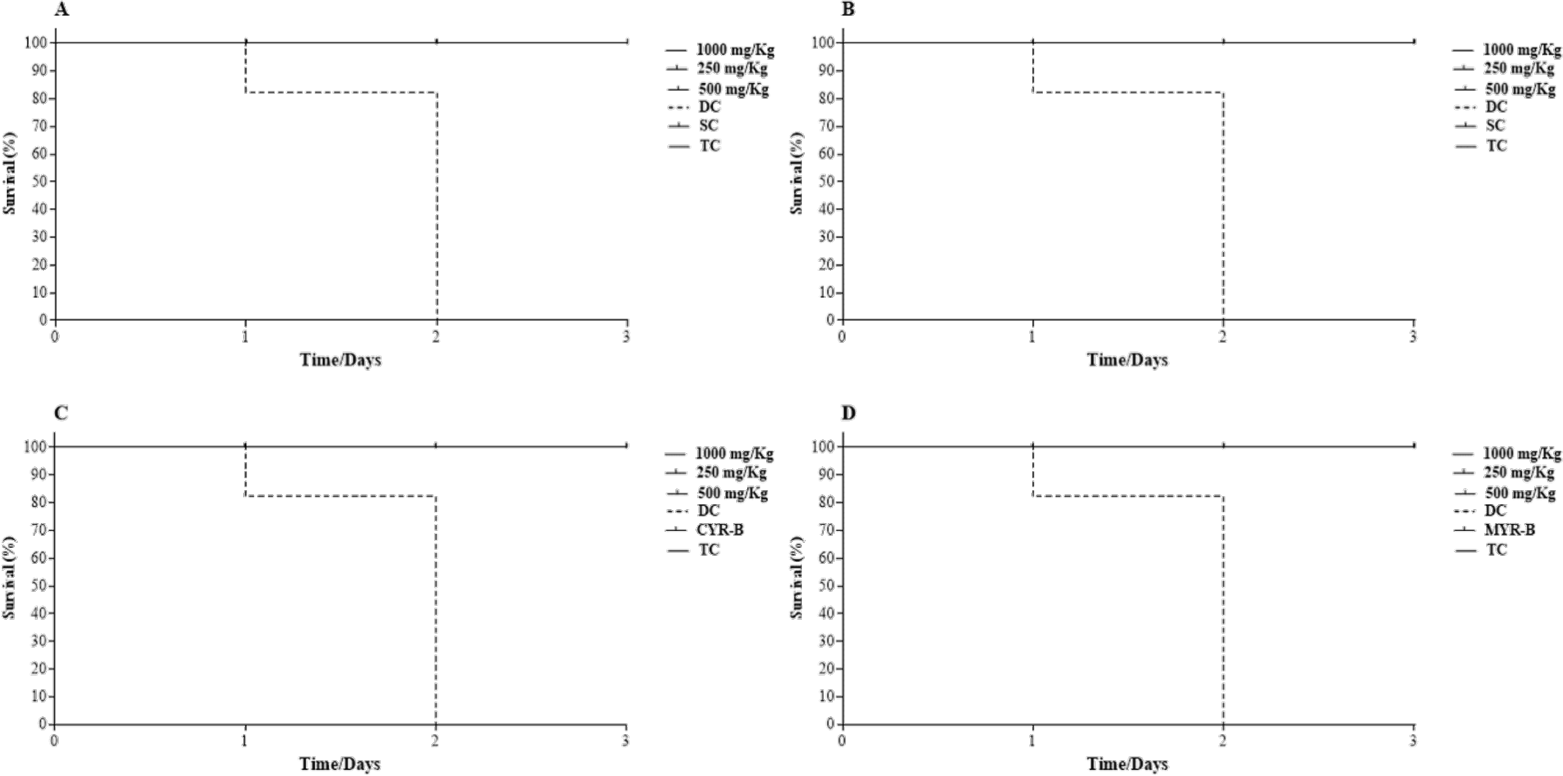
Kaplan–Meier survival curve of *G. mellonella* exposed to (A) Cym-Sol, (B) Myr-Sol, (C) Cym-NE, and (D) Myr-NE; DC: death control (100% methanol); SC: solvent control (ethanol 5%); TC: trauma control.

The in vivo acute toxicity of the NEs was assessed against *G. mellonella* larvae. No mortality was observed at concentrations ranging from 250 to 1000 mg/kg, indicating that the NEs did not cause acute toxicity (Figure 3). However, irritation was observed on day 0, as the larvae exhibited abnormal movements, such as repetitive jumping, after injection of the NEs. This behavior was not constant and ceased after 10 min. On day 1, the larvae treated with both free drugs produced a web of oily/sticky nature, particularly at higher concentrations, which persisted up to day 2. The absence of acute toxicity of nanoparticles on *G. mellonella* larvae is consistent with previous observations [53–54]. Overall, the results suggest that the NEs are not toxic to the larvae at the tested concentrations.

## Conclusion

The rHLB values for cymene and myrcene were 15 and 16, respectively. These formulations demonstrated good colloidal stability over 60 days with stable values of size, PdI, and zeta potential. In vitro release studies demonstrated that the encapsulation of myrcene or cymene in nanoemulsions led to a sustained release of the compounds, suggesting that they could potentially provide a more efficient method for delivering these compounds compared to free solutions.

Furthermore, the study showed that the nanoemulsification process reduced the cytotoxicity of terpenes, as evidenced by the lower IC_50_ values of free terpenes compared to the nanoemulsions containing monoterpenes. The in vivo acute toxicity assessment in *G. mellonella* larvae indicated that the nanoemulsions exhibit a good toxicological profile. Finally, bioassays showed that terpene nanoemulsions had equal or greater insecticidal properties than free terpenes and they might facilitate their dispersion in an aqueous environment.

The larvicidal effect of the nanoemulsions, together with their safety and sustained release attributes, holds significant promise for environmentally friendly and effective pest control. Subsequent investigations should further optimize these formulations to unlock their full potential as part of integrated pest management.

## Experimental

### Preparation of terpene nanoemulsions

Cymene (Cym-NE) and myrcene (Myr-NE) nanoemulsions were obtained by a low-energy method. Briefly, an oil phase composed of the terpene (cymene or myrcene) (5% w/w) was mixed with the surfactants (Span 80/Tween 20) (5% w/w) using a magnetic stirrer. After homogenization, the aqueous phase of ultrapure water (90% w/w) was added dropwise. The terpenes were obtained commercially from Sigma-Aldrich.

### Determination of the rHLB of the terpenes

The rHLB values of cymene and myrcene were determined by mixing different proportions of a lipophilic (sorbitan monooleate, Span 80, HLB 4.3) and a hydrophilic surfactant (polysorbate 20, Tween 20, HLB 16.7). Different formulations were prepared in a HLB range of 10.0–16.7, and the rHLB was the one in which the formulation had the best colloidal stability (Table S1, Supporting Information File 1).

### Characterization of the nanoemulsions

#### Visual appearance

The formulations obtained were maintained at room temperature and evaluated visually 24 h and 7, 14, and 21 days after preparation. Signs of instability such as creaming, sedimentation, and phase separation were recorded, as well as physical aspects such as color, transparency, and fluidity.

#### Dynamic light scattering analysis

The average hydrodynamic diameter and polydispersity index (PdI) of the NEs were evaluated over a period of 60 days using dynamic light scattering, and the zeta potential was determined via electrophoretic mobility in a Zetasizer 3000 HSA (Malvern Instruments) device, using a 10 mW HeNe laser operated at 633 nm with a detection angle of incidence of 173° at 25 °C. Data analysis was performed in automatic mode. The NEs were diluted in deionized water (1:25) before the analysis.

#### Nanoparticle tracking analysis

Nanoparticle tracking analysis was performed in a NanoSight NS300 (Malvern Instruments, United Kingdom) apparatus equipped with a sample chamber and a 638 nm laser. The samples were diluted (1:1000 v/v) in ultrapure water. The NEs were injected into the sample chamber with sterile syringes until the liquid extended to the tip of the injector. The measurements were performed in triplicate at room temperature (25 °C) and the data were represented as mean ± standard deviation.

#### Cryogenic transmission electron microscopy

The nanoemulsions were mounted onto a copper grid with lacy carbon film (300 mesh). The acquisition was carried out with a MET Talos Arctica G2 apparatus.

#### In vitro terpene release profile

The in vitro release assays were conducted assuring sink conditions. Modified Franz cells, equipped with a polyethersulfone membrane (Sigma-Aldrich) and with a diffusion area of 1.77 cm^2^ were used in the assays. A Microette (Hanson Research, USA) was used. The receptor compartment was filled with 7.0 mL of a receptor solution composed of 0.1 M phosphate buffer and ethanol (50:50 v/v), pH 5.5. 1 mL of the formulations was used, as allowed by the Franz cell.

The acceptor solution was constantly agitated at 300 rpm using mini-magnetic agitators. The temperature was maintained at 37 ± 2 °C by utilizing a circulating heating bath in the jacketed cells.

The evaluation of the release of cymene and myrcene from the nanoemulsions was performed at specific time intervals: 30 min and 1, 2, 4, 6, 8, 12, and 24 h. Each measurement was repeated six times to ensure reliability. The released compounds were quantified by high-performance liquid chromatography, following a previously validated method.

### In silico molecular and physicochemical properties of the monoterpenes

The ACD/Labs Percepta Platform, particularly the PhysChem Module, was employed to forecast molecular and physicochemical data. The ChemSpider tool facilitated the acquisition of these properties [56–57].

### Preliminary larvicidal assay

The protocol involved exposing III–IV-instar larvae to terpenes and terpene-based nanoemulsions; the mortality was recorded after 24 h. The laboratory-susceptible reference strain (Bora) from French Polynesia was utilized. The experimental protocol adhered to WHO guidelines with certain modifications [55]. Each experiment was conducted in triplicate, involving 25 third-instar larvae within each sample. Nanoemulsions diluted in distilled water at concentrations of 5, 25, and 50 mg/L were employed. For the negative control, a surfactant solution was utilized at the highest concentration of the tested samples.

### Cytotoxicity in human keratinocytes (HaCAT)

The HaCat cell line (code 341; Rio de Janeiro Cell Bank, Rio de Janeiro, Brazil) is a line of non-tumorigenic human epithelial keratinocytes. These cells were cultured in Dulbecco's modified Eagle's medium (DMEM) supplemented with 10% bovine fetal serum and 100 μg/mL of penicillin G/streptomycin. Maintained at 37 °C with 5% CO_2_, the cells were grown until they reached a subconfluent density. To detach the cells, a 5 min trypsin treatment with TrypLE™ Express at 37 °C was performed, followed by inactivation using 0.3 mg/mL trypsin inhibitor. The cells were then centrifuged at 500*g* for 5 min, resuspended in DMEM, and placed overnight in 96-well microplates (200 μL/well, 1 × 10^6^ cells/mL) at 37 °C with 5% CO_2_. After incubation, nanoemulsions, free terpenes, and surfactant solutions were administered at concentrations from 0.1 to 250 mg/mL to the cells for 24 h at 37 °C with 5% CO_2_. Cell viability was assessed using a colorimetric MTT assay. Cells were exposed to a 10 μL MTT stock solution (5 mg/mL in PBS) and incubated at 37 °C for 2 h. After incubation, the culture medium was replaced with 100 μL of DMSO. The optical density at 570 nm was measured using a microplate reader. Cell viability was determined by comparing the absorbance of each product concentration to untreated cells, with the negative control (DMEM) representing 100% cellular metabolism. The analysis utilized average values.

### In vivo toxicity evaluation

The experiment used larvae of the *G. mellonella* species, as described by Allegra et al. and Marena et al. with modifications [54,58]. A minimum of ten larvae per group (*n* = 10) were used, which were fed and raised at 25 °C until they weighed more than 0.2 mg. Larvae between 0.2 and 0.3 mg were used for the experiment, and the samples were administered (10 µL/larva) on the left side of the last proleg using a 10 µL Hamilton syringe. The larvae were then kept in the dark at room temperature and observed after 24, 48, and 72 h to evaluate their behavior, including physical aspects such as color, melanization, or loss of mobility, in response to the treatment. Death was considered when there was no physical reaction after stimulation. The samples were tested at concentrations of 250, 500, and 1000 mg/kg, with controls including trauma control (puncture only, TC), death control (100% methanol, DC), solvent control (5% ethanol, SC), and NE control (Cym-B and Myr-B).

## Supporting Information

File 1 Additional details on experimental methods and results.

## Data Availability

The data that supports the findings of this study is available from the corresponding author upon reasonable request.
