# Supplementary material for: Development and characterization of potential larvicidal nanoemulsions against Aedes aegypti
Source: Beilstein J Nanotechnol. 2024 Jan 18;15:104–14. doi: 10.3762/bjnano.15.10 (PMC10804528; doi:10.3762/bjnano.15.10)
Supplement: File 1 — Additional details on experimental methods and results. [file Beilstein_J_Nanotechnol-15-104-s001.pdf]

BEILSTEIN JOURNAL OF NANOTECHNOLOGY

Supporting Information

for

Development and characterization of potential larvicidal nanoemulsions against *Aedes aegypti*

Jonatas L. Duarte, Leonardo Delello Di Filippo, Anna Eliza Maciel de Faria Mota Oliveira, Rafael Miguel Sábio, Gabriel Davi Marena, Tais Maria Bauab, Cristiane Duque, Vincent Corbel and Marlus Chorilli

Beilstein J. Nanotechnol. **2024,** *15,* 104–114. doi:10.3762/bjnano.15.10

Additional details on experimental methods and results

License and Terms: This is a supporting information file under the terms of the Creative Commons Attribution License (https://creativecommons.org/licenses/by/4.0). Please note that the reuse, redistribution and reproduction in particular requires that the author(s) and source are credited and that individual graphics may be subject to special legal provisions.
The license is subject to the *Beilstein Journal of Nanotechnology* terms and conditions: (https://www.beilstein-journals.org/bjnano/terms)

Material and methods

Table S1: Variations in surfactant fractions to obtain different values of HLB.

rHLB	Span 80 (g)	Tween 20 (g)	Terpene (g)	Water (g)	Total (g)
10	0.27	0.23	0.5	9	10
11	0.23	0.27	0.5	9	10
12	0.19	0.31	0.5	9	10
13	0.15	0.35	0.5	9	10
14	0.11	0.39	0.5	9	10
15	0.07	0.43	0.5	9	10
16	0.03	0.47	0.5	9	10
16.7	—	0.5	0.5	9	10

Results

After 1 day

After 7 day

After 14 day

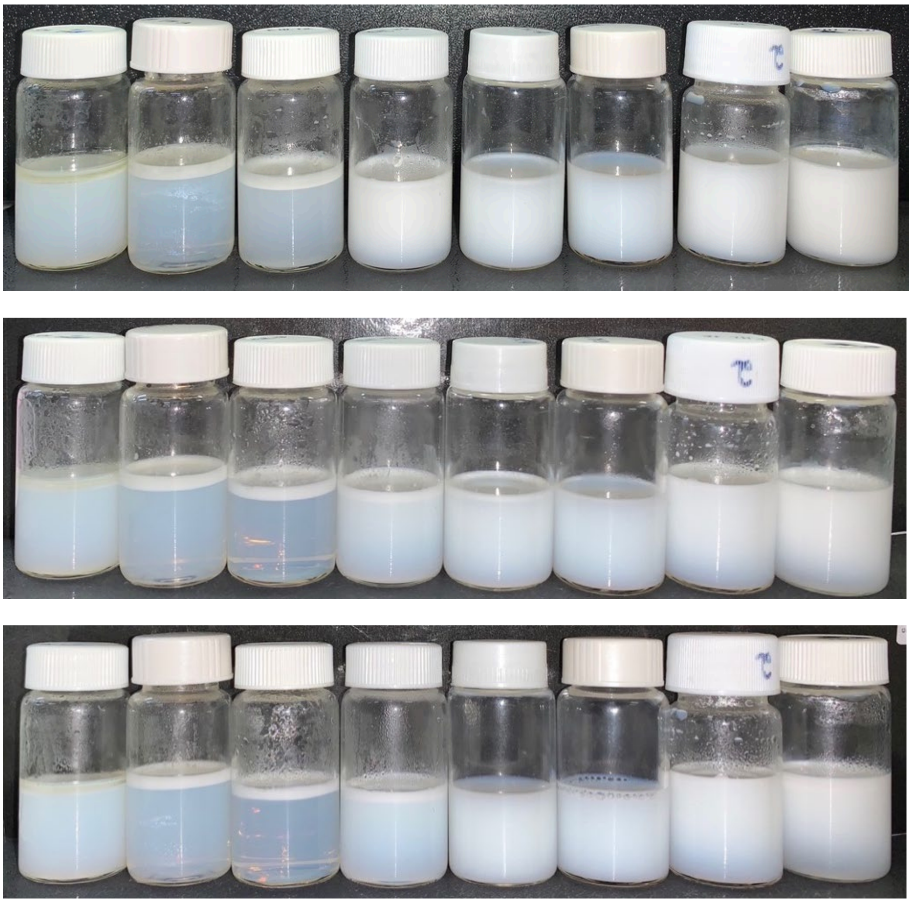

Figure S1: Visual appearance of Cym-NE on the HLB scale from 10 to 16.7.

After 1 day

After 7 days

After 14 days

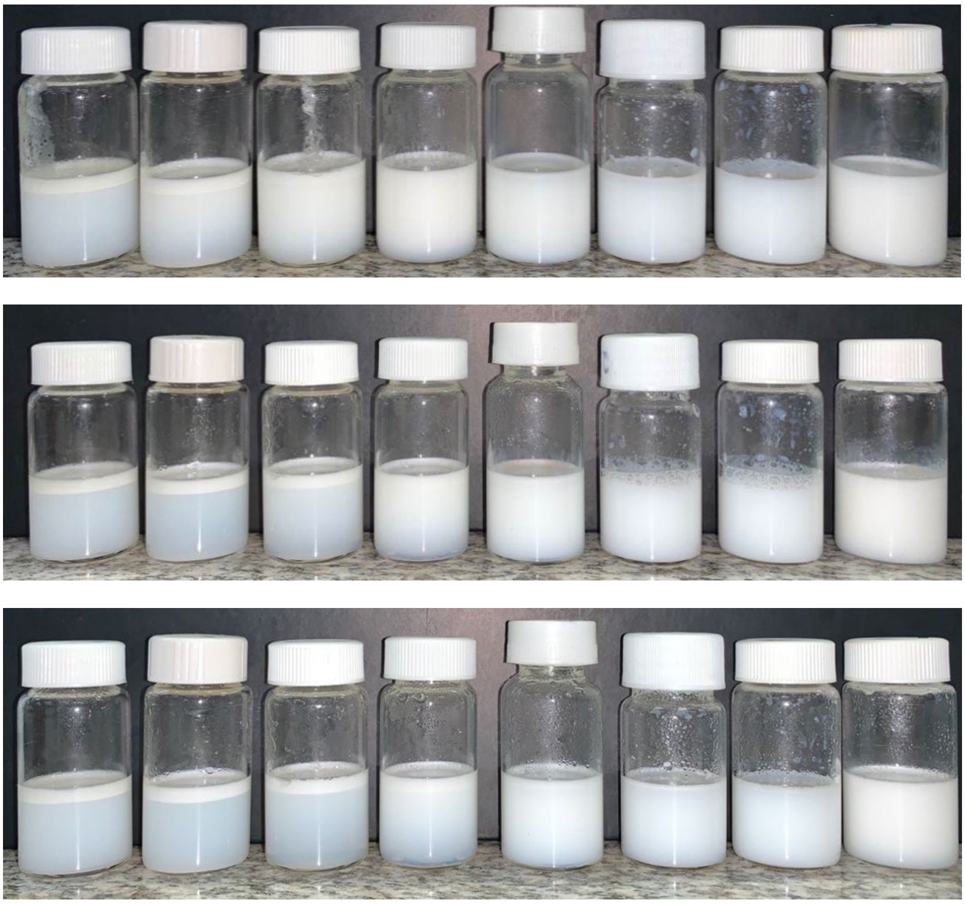

Figure 2: Visual appearance of Myr-NE on the HLB scale from 10 to 16.7.
